# Prevalence of Multiple Antibiotic Resistant Infections in Diabetic versus Nondiabetic Wounds

**DOI:** 10.1155/2014/173053

**Published:** 2014-06-25

**Authors:** Urvish Trivedi, Shamini Parameswaran, Andrew Armstrong, Diana Burgueno-Vega, John Griswold, Sharmila Dissanaike, Kendra P. Rumbaugh

**Affiliations:** ^1^Department of Surgery, Texas Tech University Health Sciences Center, MS 8312, 3601 4th Street, Lubbock, TX 79430, USA; ^2^Burn Center of Research Excellence, Texas Tech University Health Sciences Center, Lubbock, TX 79430, USA

## Abstract

Diabetes mellitus (DM) affects 23.6 million people in the USA and approximately 20–25% of diabetic patients will develop foot ulceration during the course of their disease. Up to a quarter of these patients will develop infections that will necessitate amputation. Although many studies report that the rates of antibiotic resistant infections have increased dramatically in the DM population over the last decade, to our knowledge there have been no reports directly comparing the rates of antibiotic resistant infections in DM versus non-DM wounds. We performed a retrospective study comparing the wound infections of 41 DM patients to those of 74 non-DM patients to test the hypothesis that infections with multidrug resistant organisms (MDRO) were more prevalent in the DM population. We found that 63.4% of DM and 50% of non-DM patients had MDRO infections, which was not statistically different. However, 61% of the DM patients had* Pseudomonas* infections compared to only 18.9% of non-DM patients. Furthermore, DM patients had significantly more coinfections with both* Pseudomonas* and* Staphylococcus aureus*. Though our initial hypothesis was incorrect, we demonstrated a significant correlation between* Pseudomonas* and* Pseudomonas*/*S. aureus* coinfections within DM wounds.

## 1. Introduction

Diabetes mellitus (DM) currently affects approximately 8.3% of the population and more than 79 million people have prediabetes [1]. Diabetics are more susceptible to infections due to increased glucose levels and suppressed immune response as well as the neuropathy and decreased blood flow to extremities that lead to slow-healing wounds [2]. For example, approximately 20–25% of the 23.6 million diabetics in the USA will develop foot ulceration during the course of their disease [3]. A quarter of these patients will develop infections, often with antibiotic resistant bacteria, that will necessitate amputation of their foot or leg. More than one-half of the lower extremity amputations in the USA occur among people with DM (approximately 82,000 amputations/year [4]) and are associated with significant morbidity and mortality. For example, up to 50% of patients die within the first 18 months following amputation [3, 5], and survivors face significant lifestyle impairments and frequent loss of their contralateral extremity within 5 years [6].

Amputation in diabetic patients is usually precipitated by the development of a chronic wound, clinically defined as a wound that fails to heal within 30 days [7]. Infection of these wounds, often by multidrug resistant organisms (MDRO), makes them recalcitrant to healing. Much is known about the epidemiology of diabetic chronic wound infections due to countless studies surveying the associated microorganisms. Although the specific percentages differ from study to study, the most predominant aerobes are* Staphylococcus aureus*, coagulase-negative staphylococci,* Streptococcus *species,* Enterococcus* species,* Corynebacterium* species, Enterobacteriaceae, and* Pseudomonas aeruginosa* [8]. Most of these infections are polymicrobial, and about half harbor both aerobes and anaerobes. The most predominant anaerobes are Gram-positive cocci,* Prevotella *species,* Porphyromonas *species, and* Bacteroides fragilis* [8]. An increase in the occurrence of MDRO chronic wound infections in the DM population has been noted over the last decade and has been primarily attributed to methicillin resistant* S. aureus* (MRSA), but antibiotic resistant Gram-negative organisms, particularly* P. aeruginosa*, have also been implicated [8–10].

MDRO are defined as microorganisms that are resistant to two or more classes of antimicrobial agents [11]. For diabetics, the risk factors associated with acquiring an MDRO infection have been identified and include previous antibiotic therapy and its duration, frequency of hospitalization, duration of hospital stays, osteomyelitis, and proliferative retinopathy [9, 10, 12]. These risk factors would suggest that MDRO infection rates would be higher in the diabetic population versus the nondiabetic population; however, to our knowledge, there have been no reports directly comparing the rates of MDRO infections in diabetic versus nondiabetic wounds. The goal of this study was to determine if infections with MDRO occurred more in the diabetic population, and, if so, which bacterial species were most prevalent.

## 2. Materials and Methods

The medical records of patients, aged 18–89 years, who were seen in the general surgery, vascular, trauma, and wound clinics and admitted to the University Medical Center (Lubbock, TX) with a diagnosis of chronic wound infection during the previous 10 years, were analyzed. Texas Tech University Health Sciences Center Institutional Review Board approval was obtained for collecting the data and reporting on its analyses. The database maintained information on age, sex, presence of obesity, race, hospital length of stay (LOS), disposition, and patient comorbidities (smoking, diabetes mellitus (DM), chronic kidney disease (CKD) or renal insufficiency, congestive heart failure (CHF), coronary artery disease (CAD), chronic obstructive pulmonary disease (COPD), history of vascular surgery, immunodeficiency, and previous myocardial infarction (MI)).

Any microorganism identified by the clinical laboratory during the course of the patients' wound care at University Medical Center (Lubbock, TX) and their antibiotic susceptibilities were recorded. A microbial isolate was considered to be a multidrug resistant organism (MDRO) if it was resistant to one or more classes of antimicrobial agents as determined by Clinical and Laboratory Standards Institute's antimicrobial susceptibility testing standards. Univariate analysis was performed to identify risk factors for acquiring an MDRO wound infection. Pearson correlation, Student's *t*-test, and chi-square tests were performed. All variables significant to *P* < 0.1 were then entered into a binary logistic forward-stepwise regression analysis. Variables in the multivariate analysis were also tested for significant interactions and collinearity. *P* ≤ 0.05 was considered significant on final analysis. Graphpad Prism and SPSS version 18.0 were used for analysis.

## 3. Results

A total of 115 chronic wound patients were identified (41 DM and 74 non-DM patients).  Table 1 compares the demographic information of the DM and non-DM wound groups. The DM group was significantly older and more obese than the non-DM group; however, their sex and race were comparable. Comorbidities including peripheral vascular disease (PVD), CKD/renal insufficiency, CHF, CAD, and COPD were all found significantly more frequently in the DM group.

**Figure 1:**
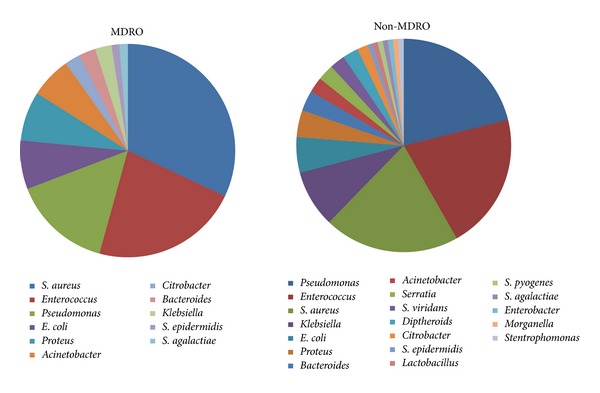
Overall prevalence of MDRO and non-MDRO isolated from wounds.

**Figure 2:**
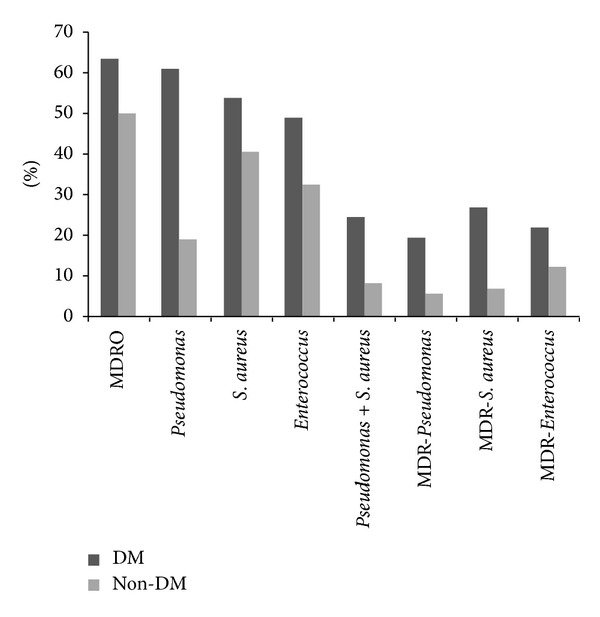
Percent of  DM and non-DM patients with MDR- and non-MDR-*Pseudomonas*, -*S. aureus,* and -*Enterococcus* wound infections.

There were 63 MDR bacterial strains isolated from the wounds included in our study.* Staphylococcus aureus* was the most prevalent MDRO, followed by* Enterococcus* and* Pseudomonas* (Figure 1). These three genera were also the most prevalent cause of non-MDRO infections, with* Pseudomonas* being number one, followed by* Enterococcus* and* S. aureus*. Out of the total 52* S. aureus* isolates, 30 (57.7%) were MRSA and 26 of these were also resistant to at least one other class of antibiotics. There were 44* Enterococcus* isolates, 18 of which were MDRO and 8 of which were vancomycin resistant (VRE). Out of the 39* Pseudomonas* isolates obtained, 12 were MDRO. Just over half of the infections were polymicrobial (52.2%) and aerobes/facultative anaerobes made up the vast majority of the bacterial population; anaerobes were only isolated from 6 wounds (all of which were* Bacteroides*). While there were more Gram-negative genera isolated from wounds, Gram-positives (e.g.,* S. aureus* and* Enterococcus*) made up a much higher percentage of the population distribution. Among the polymicrobial infections,* Pseudomonas* and* S. aureus* were most commonly found together, and coinfection was positively correlated with DM (Table 2, Figure 2).

Our hypothesis for this study was that MDRO infections were more prevalent in the chronic wounds of patients with DM. Of the total MDRO isolated 41.3% were from DM wounds (Table 2). MDRO were isolated from the wounds of 63.4% of DM patients and 50% of non-DM patients (Figure 2), which did not represent a significant correlation (chi-square, *P* = 0.258). Further, DM was not identified as a risk factor for MDRO infection with multivariate logistic regression (Table 3).

*Pseudomonas* was the most common genera of bacteria isolated from the wounds of DM patients (Figure 2).* Pseudomonas* was isolated from the wounds of 61% of DM patients but only 18.9% of non-DM patients (Figure 2), which represented a significant correlation, and this was true for MDR-*Pseudomonas* as well (Table 2).* S. aureus* and* Enterococcus* isolates were distributed more equally among the DM and non-DM population and did not correlate with either group.

The variables included and the results of the multivariate analyses are shown in Tables 3 and 4. Interestingly, smoking was the only independent risk factor identified for acquiring an MDRO wound infection (Table 3). DM was an independent risk factor for acquiring an MDR-*Pseudomonas* wound infection (*P* = 0.031) (Table 4). There was no significant interaction between variables in either multivariate model.

## 4. Discussion

The rates of chronic wound MDRO infections vary widely between institutions. The current study found a 54.8% overall prevalence of MDRO infections in the chronic wound patients admitted to our hospital over the last 10 years, which was in the midrange of the rates we have seen reported (18.2–72%, [9, 10, 13, 14]). Overall, the makeup of the microbial populations in our study also reflected those which have been frequently reported [15–17], with the most prevalent MDRO being* S. aureus* (22.6%). Also consistent with prior findings [18], Gram-positives made up the majority of isolates obtained and a little over half of the infections were polymicrobial, with* Pseudomonas* and* S. aureus* being the most commonly associated.

One notable weakness of our study, which is apparent in the data obtained, is the lack of anaerobes isolated. Anaerobic isolates were obtained from only 6 wounds, which is clearly not in line with other studies that report isolating anaerobes from approximately half of their wound cultures [15]. While the method of sampling was not determined in our study, the reliance of culturing from surface swabs still plagues many institutions even though numerous studies have demonstrated the superiority of obtaining deep tissue biopsies for culturing [19, 20]. Additionally, conventional clinical microbiology techniques, which rely on culturing, are biased for microorganisms that grow well in the laboratory (e.g., nonfastidious, aerobic microorganisms). Recent strategies that bypass culturing and identify microorganisms by DNA sequencing have revealed that up to 90% of bacteria in wounds are facultative or obligate anaerobes [21, 22], although their roles in pathogenesis and antibiotic resistance are unknown.

The goal of this study was to determine if chronic wound patients with DM contracted MDRO infections more frequently than chronic wound patients without DM. While this was not the case for MDRO in general among our patient population, we made the observation that DM was strongly correlated with* Pseudomonas* infection (both MDR and non-MDR). Others have also noted that microbial colonization of diabetic wounds appears to be different than that of nondiabetic wounds, with increased incidence of* Streptococcus*,* Staphylococcus,* and* Pseudomonas*, among others [13, 22]. Possible reasons for these differences include the altered environment of the diabetic wound (e.g., hypoxia, hyperglycemia, etc.), longer duration of infection, more frequent hospital exposures, and extended periods of antibiotic use, all of which can influence shifts in the wound microbiota. While we did not collect information concerning prior antibiotic usage, age was also identified as a risk factor in acquiring an MDR-*Pseudomonas* infection (Table 4).

Another factor that could greatly influence the ability of a wound infection to be resolved is the amount and/or type of biofilm in the wound. Biofilms are communities of microbial cells attached to a surface or each other that are surrounded by a polysaccharide matrix. It has been proposed that the presence of large amounts of biofilm in wound beds acts as a mechanical barrier to cell migration, granulation, and reepithelialization and stimulates a chronic state of inflammation, which slows healing [23–26]. It has been shown* in vitro *that bacteria residing in biofilms can be up to 1000 times more tolerant to antibiotic agents than free-floating planktonic bacteria [27]. Unlike conventional antibiotic resistance, which is typically caused by transferable genetic alterations that confer protection against antibiotics, tolerance implies a transient, nonheritable phenotype. Three main mechanisms are thought to account for bacterial tolerance within biofilms. First, the sessile or dormant state that many bacterial cells adopt when living in a biofilm makes them less susceptible to antibiotics that act only on proliferating cells. Secondly, the polysaccharide matrix surrounding the biofilm may perturb the penetration of antimicrobials, and lastly, bacterially altered microenvironments (e.g., osmotic gradients and pH differences) may antagonize antibiotic efficacy. This tolerance is completely dependent on being in the biofilm environment; thus once a microbe is removed (e.g., by swab culture) and proliferated* in vitro*, subsequent generations of planktonic cells will revert back to their susceptible phenotypes. Thus, even if a clinical isolate does appear to be an MDRO via conventional susceptibility testing, this does not mean that it does not display biofilm-related tolerance* in vivo*.

In our study biofilm formation may provide an explanation as to why smoking was identified as a risk factor for acquiring an MDRO infection (Table 3). While smoking causes many alterations to the host immune response that can predispose smokers to infection and/or delayed wound healing [28, 29], we found it puzzling that it was not also identified as a risk factor for* Pseudomonas* infection. One possible explanation for this finding is that the majority of MDRO infections in our study were caused by* S. aureus, *a microorganism notorious for causing biofilm-related infections [30]. Recently it was shown that cigarette smoke increased the ability of* S. aureus* to adhere to host cells and form biofilms* in vitro* [31]. While this study was conducted using human upper airway epithelial cells, in an attempt to explain high levels of upper respiratory infections in smokers, it is possible that smoking increases the likelihood of* S. aureus* adhering and forming biofilms in the wounds of smokers.

Host environmental conditions also greatly affect the ability of* P. aeruginosa* to form biofilms. We recently showed that* P. aeruginosa* biofilm formation was enhanced in the wounds of diabetic mice compared to their nondiabetic littermates, especially when the diabetic mice were on insulin therapy. These significantly elevated amounts of biofilm resulted in increased antibiotic tolerance and delays in healing [32]. While the mechanisms involved are still not completely understood, we showed that insulin stimulated* P. aeruginosa* cells to adopt a biofilm lifestyle and that insulin treatment modulated the diabetic immune system to favor* P. aeruginosa* biofilm formation [32, 33]. Thus, based on these findings, it is reasonable to speculate that* P. aeruginosa* is more prevalent in diabetic wounds not only because diabetic patients simply come into contact with it more frequently, but also because there is some selective pressure in the environment of the diabetic wound that favors* Pseudomonas* colonization and/or persistence.

## Figures and Tables

**Table 1:** Demographics and comorbidities associated with the wound population surveyed.

	Total	DM	Non-DM	*P* value
*N*	115	41	74	
Age	48.8 ± 16.9 (11–86)	55 ± 14.8 (33–86)	45.4 ± 17.1 (11–81)	0.0029
% obese BMI	42.6	61	32.4	0.0028
% male	54.8	43.9	60.8	NS
Race	59-W, 8-B, 40-H, 8-U	20-W, 2-B, 18-H, 1-U	39-W, 6-B, 22-H, 7-U	NS
Hospital LOS (days)	17 ± 36.7 (0–358)	13 ± 13.2 (0–50)	19.3 ± 45 (0–358)	NS
PVD	22.6	41.5	12.2	0.0002
Smoker	32.2	41.5	27	NS
CKR/renal insuf.	14.8	29.3	6.8	0.0010
CHF	15.7	31.7	6.8	0.0003
Previous MI	5.2	4.9	5.4	NS
CAD	11.3	19.5	6.8	0.0388
CVA	5.2	9.8	2.7	NS
COPD	8.7	17.1	4.1	0.0175
Hx of vascular surgery	8.7	12.2	6.8	NS
Immunodeficiency	2.6	0	4.1	NS

*N* is the number of patients. Age is shown as the mean, standard deviation and range. Sex is expressed as the percentage male. BMI is expressed as percent within obese range. Race is given by number of patients who were white (W), black (B), or Hispanic (H) or whose race was not given (U). Comorbidities are expressed as a percentage of the DM or non-DM population. Two-tail *P* value was determined with Pearson correlation coefficients and indicates that the variable significantly correlates with DM. NS: not significant.

**Table 2:** Bacteria isolated from wounds.

Bacteria isolated	DM	Non-DM	*P* value
*Staphylococcus aureus *	42.3 (22/52)	57.7 (30/52)	NS
*Pseudomonas *	64 (25/39)	35.9 (14/39)	<0.0001
*Enterococcus *	45.5 (20/44)	54.5 (24/44)	NS
*Pseudomonas* and * S. aureus* coinfection	62.5 (10/16)	37.5 (6/16)	0.016
MDRO	41.3 (26/63)	58.7 (37/63)	NS
MRSA	50 (15/30)	50 (15/30)	NS
MDR-*S. aureus *	42.3 (11/26)	57.7 (15/26)	NS
MDR-*Pseudomonas *	66.7 (8/12)	33.3 (4/12)	0.018
MDR-*Enterococcus *	50 (9/18)	50 (9/18)	NS

Values are expressed as a percentage of the total number of isolates in the DM or non-DM population. The numbers in parenthesis are number of isolates from DM or non-DM divided by total number of isolates obtained from all wounds. Two-tail *P* value was determined with Pearson correlation coefficients and indicates that the variable significantly correlates with DM. NS: not significant.

**Table 3:** Multivariate analysis (*P* value) of risk factors related to MDRO infection.

Variable	Significance	95% confidence interval	
		Lower	Upper
Gender	0.068	0.946	4.751
Smoker	0.041	0.153	0.961
Previous MI	0.080	0.775	97.955
Hospital LOS	0.152	0.993	1.043

**Table 4:** Multivariate analysis (*P* value) of risk factors related to MDR-*Pseudomonas* infection.

Variable	Significance	95% confidence interval	
		Lower	Upper
Age	0.040	0.888	0.997
DM	0.035	1.132	30.312
Smoker	0.753	0.277	5.882
CHF	0.207	0.044	6.480
CVA	0.622	0.033	3.106
COPD	0.325	0.033	3.106
